# Vitamin D Binding Protein and Renal Injury in Acute Decompensated Heart Failure

**DOI:** 10.3389/fcvm.2022.829490

**Published:** 2022-06-09

**Authors:** Elisa Diaz-Riera, Maisa García-Arguinzonis, Laura López, Xavier Garcia-Moll, Lina Badimon, Teresa Padró

**Affiliations:** ^1^Cardiovascular-Program ICCC, Research Institute–Hospital Santa Creu i Sant Pau, IIB-Sant Pau, Barcelona, Spain; ^2^Faculty of Medicine, University of Barcelona (UB), Barcelona, Spain; ^3^Cardiology Department, Hospital Santa Creu i Sant Pau, Barcelona, Spain; ^4^Centro de Investigación Biomédica en Red Cardiovascular (CIBERCV), Instituto de Salud Carlos III, Madrid, Spain; ^5^Cardiovascular Research Chair, Universitat Autónoma de Barcelona (UAB), Barcelona, Spain

**Keywords:** renal dysfunction, vitamin D binding protein (GC), urine sample analysis, heart failure, proteomics, two dimension electrophoresis–MS/MS

## Abstract

**Background:**

Renal function in acute decompensated heart faiulre (ADHF) is a strong predictor of disease evolution and poor outcome. Current biomarkers for early diagnostic of renal injury in the setting of ADHF are still controversial, and their association to early pathological changes needs to be established. By applying a proteomic approach, we aimed to identify early changes in the differential urine protein signature associated with development of renal injury in patients hospitalised due to ADHF.

**Materials and Methods:**

Patients (71 [64–77] years old) admitted at the emergency room with ADHF and hospitalised were investigated (*N* = 64). Samples (urine/serum) were collected at hospital admission (day 0) and 72 h later (day 3). Differential serum proteome was analysed by two-dimensional electrophoresis and matrix-assisted laser desorption/ionisation-time of flight (MALDI-ToF/ToF). Validation studies were performed using ELISA.

**Results:**

Proteomic analysis depicted urinary vitamin D binding protein (uVDBP) as a two spots protein with increased intensity in ADHF and significant differences depending on the glomerular filtration rate (GFR). Urinary VDBP in patients with ADHF at hospitalisation was > threefold higher than in healthy subjects, with the highest levels in those patients with ADHF already presenting renal dysfunction. At day 3, urine VDBP levels in patients maintaining normal renal function dropped to normal values (*P* = 0.03 vs. day 0). In contrast, urine VDBP levels remained elevated in the group developing renal injury, with values twofold above the normal range (*P* < 0.05), while serum creatinine and GF levels were within the physiological range in this group. Urinary VDBP in ADHF positively correlated with markers of renal injury such as cystatin C and Kidney Injury Molecule 1 (KIM-1). By ROC analysis, urinary VDBP, when added to cystatin C and KIM-1, improved the prediction of renal injury in patients with ADHF.

**Conclusion:**

We showed increased urine VDBP in patients with ADHF at hospital admission and a differential uVDBP evolution pattern at early stage of renal dysfunction, before pathological worsening of GFR is evidenced.

## Introduction

Renal insufficiency is present as comorbidity in nearly a quarter of patients hospitalised due to acute decompensated heart failure (ADHF) (1, 2), and the evolution of renal function has prognostic relevance in these patients (2). Indeed, ADHF is frequently complicated by acute kidney injury during hospitalisation (2, 3), which is associated with adverse outcomes and longer hospital stays (2, 4, 5). In this respect, a recent meta-analysis indicated that all-cause mortality increased significantly in patients with ADHF compared with those without acute kidney injury (5). The complex relationship between heart failure and renal dysfunction may result in a combination of hormonal, hemodynamic, and inflammatory factors (6) and aggressive decongestive treatment (7). In turn, kidneys are responsible for sodium homeostasis playing a key role to control intravascular and interstitial fluid accumulation (congestion) in heart failure (8). Until now, however, the mechanisms participating in the cardiorenal axis pathology need to be better understood, and the early differential protein pattern associated to these processes remains to be identified.

Deterioration of kidney function is frequently recognised by the rise of serum creatinine levels or by the decline of glomerular filtration rate (GFR). However, creatinine levels can also be influenced by several factors such as advanced age, sex, excessive protein intake, muscle mass, medication, or intense exercise (9). Nowadays, early changes in other molecular components have been suggested including kidney injury molecule 1 (KIM-1), Cystatin C (CysC), or neutrophil gelatinase-associated lipocalin (NGAL) (10–13), but their usefulness for detecting early pathological changes is controversial, and their value has not been consistently proven (14, 15). Therefore, identification of molecular components that accurately facilitate early detection of renal injury and may contribute to a better stratification of patients with ADHF and enhance their management remains a medical challenge.

Over the last decade, urinary proteome has become a potential source of novel disease molecular patterns and urinary proteomic analysis in heart failure has been used to identify the differential expression profile associated to earlier diagnosis of the disease (16–18) and to establish diagnostic classifiers in patients with heart failure with specific disease conditions (19).

In this study, we applied a mass spectrometry-based protein discovery approach (20, 21) aimed to identify urinary proteins associated with renal dysfunction in hospitalised patients with ADHF. By proteomics, we have identified significant changes in vitamin D binding protein (VDBP), a member of the albumin superfamily of binding proteins that is produced in the liver, filtered through the glomerulus as a complex with vitamin D3 (25[OH]D3) and actively reabsorbed in the proximal tubule cells (22, 23).

Anomalous urinary loss of VDBP, due to impaired proximal tubular reabsorption, has been reported in the setting of renal damage in preclinical studies and metabolic disease-related nephropathies in humans (24, 25). In this hypothesis-generating study, we analysed urinary VDBP levels in association with renal function in patients with ADHF during the first 72 h hospitalisation after an acute event to investigate the value of VDBP to provide accurate pathophysiologic information on kidney function deterioration in ADHF before creatinine levels rise.

## Materials and Methods

### Study Population and Study Design

**FIGURE 1:**
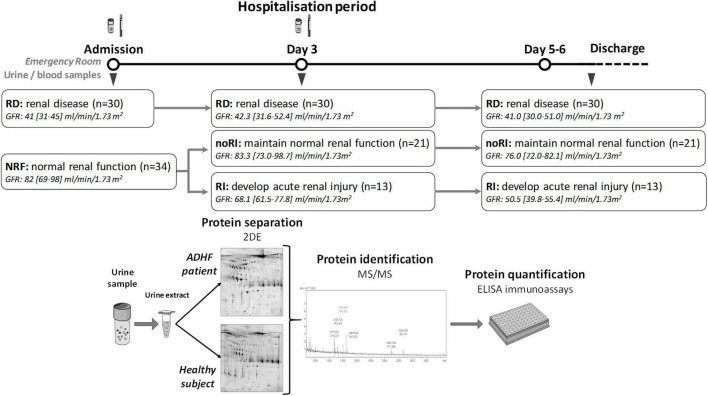
Study design. Patients with ADHF were divided in two groups at hospital admission depending on their glomerular filtration rate. RD group included patients with glomerular filtration rate <60 ml/min/1.73 m^2^, while NRF presented glomerular filtration rate above this cutoff. At day 3, patients with NRF were divided in two groups depending on their kidney function evolution. RI patients developed renal function deterioration defined by glomerular filtration rate <60 ml/min/1.73 m^2^, while noRI patients maintained normal glomerular filtration rate. Urine and blood samples were obtained at hospital admission and at day 3 of hospitalisation. Protein extracts from urine were prepared, and samples were analysed by two-dimensional (2DE)-electrophoresis and mass spectrometry for identification of differential protein patterns. Validation studies were performed by ELISA. Sample size is given in Figure.

This study refers to patients with ADHF that were hospitalised in Hospital de la Santa Creu i Sant Pau (HSCSP), in Barcelona, between February 2017 and March 2020. Patients hospitalised with ADHF were distributed in two different groups depending on their kidney function at admission. Renal function was shown as MDRD-4 (ml/min/1.73 m^2^), a serum creatinine-based estimation obtained using clinical data of the patients. Levels below 60 ml/min/1.73 m^2^ were considered pathological. Two groups were formed: I) patients with ADHF with renal dysfunction (RD) at hospital admission and II) patients with ADHF with normal renal function (NRF) at hospital admission. From this latter NRF group, two further subgroups were made based on the evolution of the renal function during hospitalisation. Patients who developed renal function deterioration to pathological levels (RI-Group; MDRD-4 < 60 ml/min/1.73 m^2^) and those who maintained normal renal function (noRI). A group of healthy subjects (HS, *N* = 13, 50 [37–63] years) was used to establish the normal range in urine of the identified proteins (Figure 1).

The Ethics Committee of the Santa Creu i Sant Pau Hospital in Barcelona, Spain, approved this study, and it was performed according to principles of Helsinki’s Declaration. All patients signed an informed consent prior being included in the study. Patients under chemotherapy, pregnancy, or postdelivery ischemic heart syndrome in women and other causes of acute episode (myocardial infarction, myocarditis, or toxic aetiology) were excluded from the study. Medication was not considered as exclusion criteria except those required in oncological treatment (who were already excluded).

### Biological Samples

Urine and blood samples were collected at hospital admission and at day 3 of hospitalisation, when patients no longer showed signs of dehydration due to the acute event. Samples at hospital admission were collected as soon as possible after hospitalisation. Urine obtained at 72 h refers to the first urine in the morning and blood extraction. Blood extraction at day 3 was performed at the same time of urine collection. All samples were processed identically and within 30 min after obtention. Protease inhibitors (cOmplete Mini, Roche, Basel, Switzerland) were added to urine samples at collection. Urine samples were centrifuged (1,200 *g*, 10 min, room temperature) to precipitate debris, aliquoted, and stored at –80^°^C until further analysis. The level of total protein in urine was analysed with a Clima MC-15 analyser using the specific Gernon kit (RAL S.A., Barcelona, Spain), as described by the providers.

Serum was obtained after maintaining blood samples for 30 min at 37^°^C followed by 30 min at 4^°^C. Thereafter, samples were centrifuged at 1,800 *g* (30 min, 4^°^C), aliquoted, and stored at –80^°^C until analysed.

Blood creatinine (Jaffe reaction), NT-proBNP (electroquimioluminiscence), urea (kinetic urease), and haemoglobin were analysed by standard laboratory methods as part of the patients’ routine analyses. Glomerular filtrate was calculated using the MDRD-4 algorithm that includes patient’s plasma creatinine levels, age, sex, and race (26). Total protein and creatinine in urine were analysed in a Clima MC-15 analyser using Gernon kits for creatinine and total protein (RAL S.A., Barcelona, Spain).

Urine samples for the HS reference group were collected in the morning, and samples were processed as described for the patients with ADHF.

### Two-Dimensional Electrophoresis and Mass Spectrometry

The analysis of differential protein patterns was performed by a proteomic approach using two-dimensional electrophoresis (2DE) followed by mass-spectrometry for protein identification, as previously described (27).

Urine samples (4 ml) of patients with ADHF and healthy controls were concentrated and desalted by centrifugation (3,220 *g*, 30 min, and 10^°^C) using 3 kDa cut-off filter devices (Amicon Ultra-4, Millipore, Burlington, MA, United States) and 100 mM Tris-HCl, pH 7.6. A final volume of 1 ml was obtained and depleted of albumin and IgGs using the ProteoExtract Albumin/IgG Removal Kit (Calbiochem, San Diego, CA, United States), as reported by the providers. Thereafter, sample buffer was exchanged to a urea-containing buffer (7 M urea, 2 M thiourea, 2% CHAPS), by centrifugation with the 3 kDa cut-off filter devices (3,220 *g*, at room temperature) until a final volume of 400 μl was obtained. Protein concentration in urine extracts was measured with 2D-Quant Kit (GE Healthcare, Chicago, IL, United States), and 100 μg total protein aliquots were prepared.

A 100 μg aliquot was used to perform the individual 2DE analytical gels and 300 μg for the preparative gels (pool from five representative samples), which refer to, approximately, 1 and 3 ml, respectively, of original urine samples. Each aliquot of the urea/thiourea/chaps urine extracts was applied to 17 cm dry strips (ReadyStrips IPG strips, pH 4–7 linear range; BioRad, San Diego, CA, United States), using the PROTEAN i12 IEF system (Bio-Rad, San Diego, CA, United States) for the first dimension, as previously described in our group (28, 29). The second dimension was resolved in 12% SDS-PAGE. Gels were fixed for 2 h (40% ethanol and 10% acetic acid), developed with Flamingo (Bio-Rad, San Diego, CA, United States) for protein fluorescent staining. Protein spot quantification and analysis for differences between gels was performed using the PDQuest analysis software (Bio-Rad, San Diego, CA, United States). Each spot was assigned a relative value (AU) that corresponds to the single spot volume compared with the volume of all spots in the gel, following background extraction and normalisation between gels, as previously reported (28). This software specifically analyses differences in protein patterns, in which a master gel is created wherein all gels are included and used to compare with each individual sample.

Proteins were identified after in-gel tryptic digestion and extraction of peptides from the gel pieces by matrix-assisted laser desorption/ionisation time-of-flight (MALDI-TOF) using an AutoFlex III Smart beam MALDI-TOF/TOF (BrukerDaltonics, Billerica, MA, United States), as previously described (28). Samples and calibrants were mixed 1:1 with alpha-cyano-4-hydroxycinnamic acid (HCCA) matrix (0.7 mg/ml) and were applied to Anchor Chip plates (BrukerDaltonics, Billerica, MA, United States).

Spectra were acquired with flexControl on the reflectron mode (mass range m/z 850–4,000; reflectron 1, 21.06 kV; reflectron 2, 9.77 kV; ion source 1 voltage, 19 kV; ion source 2, 16.5 kV; detection gain, 2.37×) with an average of 3,500 added shots at a frequency of 200 Hz. Samples processed with flexAnalysis (version 3.0, Bruker Daltonics, Billerica, MA, United States) considering a signal-to-noise ratio > 3, applying statistical calibration, and eliminating background peaks. After processing, spectra were sent to the interface BioTools (version 3.2, Bruker Daltonics, Billerica, MA, United States), and MASCOT search on Swiss-Prot 57.15 database was carried out [taxonomy, *Homo sapiens*; mass tolerance, 50–100; up to two trypsin miss cleavages; global modification: carbamidomethyl (C); and variable modification: oxidation (M)]. Identification was carried out by peptide mass fingerprinting (PMF) where a MASCOT scores > 56 and at least five matched peptides was accepted. Confirmation of identified protein was performed by peptide fragmentation working on the LIFT mode (MS/MS) (27, 28).

### Western Blot

Urinary protein extracts were resolved by 1-DE under reducing conditions and electrotransferred to nitrocellulose membranes. Urinary VDBP detection was performed using a rabbit monoclonal antibody (ab81307, 1:5,000 in 5% NFDM-TBST, Abcam, Cambridge, United Kingdom). Band detection was performed using chemiluminescent SuperSignal (FischerScientific, Waltham, MA, United States) and a molecular imager ChemiDoc XRS System, Universal Hood II (BioRad, San Diego, CA, United States).

### Immunoassays

Vitamin D binding protein was analysed in urine and serum samples using Human Vitamin D BP Quantikine ELISA kit (DVDBP0B, R&D Systems, Minneapolis, MN, United States) with a sensitivity of 0.338 ng/ml and with intraassay variability < 2.5% and variability <7% in interassay trials. Urinary Cystatin C (uCysC) levels were analysed using Human Cystatin C ELISA kit from Biovendor (RD191009100, Biovendor, Brno, Czech Republic), with a sensitivity of 0.25 ng/ml, intraassay variability < 4%, and with interassay variability < 11%. Urinary KIM-1 levels were analysed using Human KIM1 ELISA Kit (ab235081, Abcam, Cambridge, United Kingdom) with a sensitivity of 1.28 pg/ml and with intraassay variability <3% and interassay variability <7%. Each immunoassay was performed according to the providers’ protocol. Concentrations of the proteins in urine were expressed per total protein content in the same sample to correct for difference in total protein concentration.

### Statistical Analysis

Data were expressed as median and interquartile range (IQR). N indicates the number of subjects tested. The normal distribution was determined *via* the Kolmogorov-Smirnov test. Statistic differences between groups for non-normally distributed continuous variables were analysed by non-parametric tests, including Mann-Whitney or Kruskal-Wallis tests. Frequencies of categorical variables were compared by Chi-square analyses. Correlations between variables were determined using single regression models and Spearman rank correlation. The Wilcoxon Signed Rank test was used to compare each patient evolution. Due to the exploratory character of this proteomic study, determination of the sample size was based on past experience with similar studies (28). Data of patients with ADHF at hospital admission obtained from the proteomic studies served to check sample size in the validation quantitative analysis (i.e., ELISA method). Minimal required sample size was calculated and validated using the JavaScript-based method for simple power and sample size calculation when two independent groups are compared, provided in http://www.stat.ubc.ca/~rollin/stats/ssize/n2.html (28). Specifically, based on the median value of the ADHF group (at hospitalization) and the variability range of the total population, a N = 64 in the patient group results in a study power >0.75 (type I error = 0.05, two-sided test). Receiver operating characteristic (ROC) curve analyses were used to calculate the area under the curve for each variable along with its 95% CI and to determine the power to discriminate renal function deterioration. The statistical analysis was performed using Stata v15 and SPSS v26. A *P*-value ≤ 0.05 was considered statistically significant.

## Results

### Clinical Characteristics of the Acute Decompensated Heart Failure Patient Population

Baseline demographic, clinical characteristics, and background medication of the studied population are given in Table 1. Briefly, this refers to 64 patients with ADHF with median age of 71[64–77] years and with 68% men, who were hospitalised at Hospital de la Santa Creu i Sant Pau (HSCSP), Barcelona. At the time of hospitalisation, 47% of patients with ADHF presented pathological GFR levels (RD group; MDRD-4: 41.1 [30.7–45.3] ml/min/1.73 m^2^, *P* < 0.001) while the other 53% of ADHF (NRF group) presented GFR levels within the normal range with MDRD-4 > 60 ml/min/1.73 m^2^ (82.3 [69.0–98.3] ml/min/1.73 m^2^). Notably, 38% of patients with ADHF within the NRF group (normal kidney function at hospital admission) showed renal function deterioration with GFR values below the pathological cut-off (60 ml/min/1.73 m^2^), which referred to a mean increase of 45% of serum creatinine, after day 5 of hospitalisation (Group RI; see Figure 1).

**TABLE 1:** Patient baseline characteristics.

	All patients *N* = 64	NRF *N* = 34	RD *N* = 30	*P*-value
**Demographic characteristics; median [IQR]**				
Female/male, N	20/44	9/25	11/19	0.146
Age, years	71.0 [64.0–77.0]	59.0 [58.0–75.0]	74.5 [69.0–78.0]	**0.010**
Weight, kg	72.6 [61.2–86.6]	70.0 [59.8–86.8]	75.8 [62.0–84.0]	0.591
**Kidney function markers**				
Creatinine, μmol/L	104.5 [78.0–146.3]	78.5 [67.8–96.8]	147.0 [120.8–195.5]	**<0.001**
Glomerular filtration (MDRD-4)	61.3 [41.8–83.1]	82.3 [69.0–98.2]	41.1 [31.0–45.3]	**<0.001**
Urea, mmol/L	10.1 [7.0–15.6]	7.1 [5.8–9.2]	16.5 [12.9–23.0]	**<0.001**
**Cardiac function markers**				
NT-proBNP, μg/L	4.14 [2.5–8.9]	3.4 [1.9–6.4]	5.2 [3.0–14.7]	**0.022**
Left ventricular ejection fraction (LVEF),%	45.5 [33.0–57.5]	37.5 [33.0–57.0]	51.0 [33.0–58.0]	0.297
Preserved LVEF, N (%)	27 (42)	11 (32)	16 (53)	
Reduced LVEF, N (%)	27 (42)	18 (53)	9 (30)	0.158
Mid-range LVEF, N (%)	10 (16)	5 (15)	5 (17)	
Atrial fibrillation, N (%)	31 (48)	16 (47)	15 (50)	>0.999
Cardiovascular disease, N (%)	20 (31)	10 (29)	10 (33)	0.791
**Other biochemical markers**				
Haemoglobin, g/L	122 [104–138]	129 [114–141]	113 [96–125]	**0.008**
**Risk factors; N (%)**				
Active smoking	10 (16)	8 (24)	2 (7)	0.088
Hypertension	47 (73)	21 (62)	26 (87)	**0.046**
Pulmonary hypertension	16 (25)	8 (24)	8 (27)	0.781
Diabetes mellitus type 2	28 (44)	11 (32)	17 (57)	0.079
Dyslipidaemia	44 (69)	20 (59)	24 (80)	0.108
**Background medication; N (%)**				
Diuretics	47 (73)	21 (59)	27 (90)	0.011
Statins	41 (64)	18 (53)	23 (77)	0.068
Anticoagulants	26 (41)	14 (41)	12 (40)	>0.999
Antiplatelet agents	36 (56)	19 (56)	17 (57)	>0.999
Beta-blockers	44 (69)	21 (62)	23 (77)	0.281
Antiarrhythmic agents	6 (9)	4 (12)	2 (7)	0.676
Antidiabetics	24 (38)	8 (21)	17 (57)	**0.010**
Insulin	11 (17)	2 (6)	9 (30)	**0.018**
Oral antidiabetic agents	21 (33)	8 (24)	13 (43)	0.114
ACE inhibitor/ARB	43 (67)	26 (76)	17 (57)	0.114

*P-values compare NRF and RD patients, and it is calculated using the Fisher exact test, except for LVEF where χ^2^ was used. Diuretics: hydrochlorothiazide, furosemide, eplerenone, and spironolactone. Statins: atorvastatin, pravastatin, simvastatin, ezetimibe. Anticoagulants: warfarin, acenocoumarol, bemiparin, heparin, dabigatran, rivaroxaban, edoxaban, and apixaban. Antiplatelet agents: acetylsalicylic acid and clopidogrel. Beta-blockers: bisoprolol and carvedilol. Antiarrhythmic agents: amiodarone. Oral antidiabetic agents: metformin and repaglinide. Angiotensin-converting enzyme inhibitors (ACEIs) include: captopril, enalapril, and ramipril. Angiotensin receptor blockers (ARBs): losartan, olmesartan, and valsartan.*

*Bold values emphasize a statistically significant result (p < 0.05).*

All patients with ADHF had plasma NT-proBNP values above the pathological cut-off (1.8 μg/L), being levels in the RD group significantly higher than those in the NRF group (5.2 [3.0–14.7] μg/L vs. 3.4 [1.9–6.4] μg/L, *P* = 0.022). Forty-two percent of patients with ADHF had reduced left ventricular ejection fraction (LVEF; 32 [21–35]%), and 42% had an LVEF above 50% (60 [56–61]%) at admission. The remaining group of patients (16%) presented mid-range LVEF, with values between 40 and 49% (46 [44–47]%). Mean hospitalisation time was 10.8 ± 5.3 days for all patients; however, a longer trend was observed in the subgroup of patients developing RI compared with those who maintained normal renal function (12 [9–17] days; 9 [7–11] days, respectively, *P* = 0.075).

Regarding medication, 73% of our study group were already under diuretic treatment before hospital admission. Upon hospitalisation due to ADHF, patients are given intravenous furosemide (dosage depending on their clinical condition, prior diuretic dosage and renal function) until stabilisation for decongestion. Later, patients were administered furosemide orally and complemented with other diuretics if needed, and the dose was dependent on the patients’ condition and response to avoid increases in serum creatinine.

A representative subgroup of 17 patients with ADHF (76% men, 72 [69–76] years old), with renal dysfunction (RD, *N* = 5), and with normal renal function (NRF, *N* = 12) at hospitalisation was used for proteomic studies in an initial discovery phase. Demographic and clinical characteristics of this subgroup are given in Supplementary Table 1.

### Urine Differential Proteomic Profile in Patients With Acute Decompensated Heart Failure

The urinary proteome of patients with ADHF at hospital admission was compared with that of a healthy control group in the discovery phase. A total of 23 differential proteins were detected, with varying functions (cell signalling, cell metabolism, coagulation, acute phase response), as shown in Supplementary Figure 1. Three differential proteins were associated to vitamin metabolism and identified as transthyretin (TTR, Swiss-Prot P02766, Mascot-score 61), retinol binding protein 4 (RBP4, Swiss-Prot P02753, Mascot-score 66), and VDBP (Swiss-Prot P21614, Mascot-score 72) (Supplementary Table 2).

**FIGURE 2:**
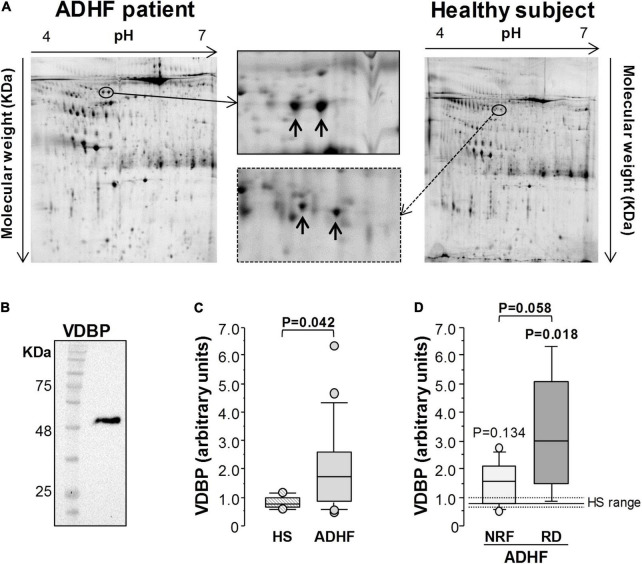
Proteomic analysis of urine samples. **(A)** Representative 2-DE image of human urine samples in a pI range of 4–7 and 12% SDS-PAGE gels. Vitamin D binding protein (VDBP) was identified as 2 spots (pI: 5.25–5.30; Mw: 50.3 KDa) in patients with ADHF (*N* = 17) and healthy subjects (*N* = 6). **(B)** Western blot analysis showed one single band for VDBP in urine samples. **(C,D)** Box plot diagrams [median (IQR)] of the intensity of both VDBP spots at hospital admission. In **(C)**, comparison between patients with ADHF and heathy subjects (HS). **(D)** Differences in VDBP intensity between patients with ADHF with (*N* = 12) and without (*N* = 5) renal dysfunction (RD and NRF for GFR values below and above 60 ml/min/1.73 m^2^, respectively). The Mann-Whitney test was used for comparison between groups in C and D. Statistical significance for *P* < 0.05.

Vitamin D binding protein in urine was identified by 2DE-MS/MS as two spots with pI within 5.25–5.30 and a molecular weight of 50.3 KDa (Figure 2A and Supplementary Figure 2). By Western blot analysis, VDBP was found as a single band in urine samples of patients with ADHF (Figure 2B). Compared with healthy subjects, patients with ADHF at hospital admission showed a twofold higher urinary levels (ADHF, *N* = 17, 1.71 [0.87–2.55] AU vs. 0.77 [0.66–0.98] AU of HS, *N* = 6, *P* = 0.042, Figure 2C). Within patients with ADHF at hospitalisation, the RD group (*N* = 5, 2.98 [1.71–4.66] AU) presented the highest values; these being twofold higher than those of the NRF group (*N* = 12, 1.58 [0.76–2.12] AU, *P* = 0.058) (Figure 2D).

While no differences were observed between patients depending on presence of atrial fibrillation (*P* = 0.228), prior cardiovascular disease (*P* = 0.428), hypertension (*P* = 0.113), dyslipidaemia (*P* = 0.833), or diabetes mellitus (DM2, *P* = 0.228).

### Increased Levels of Urinary Vitamin D Binding Protein in Patients With Acute Decompensated Heart Failure at Hospital Admission

**FIGURE 3:**
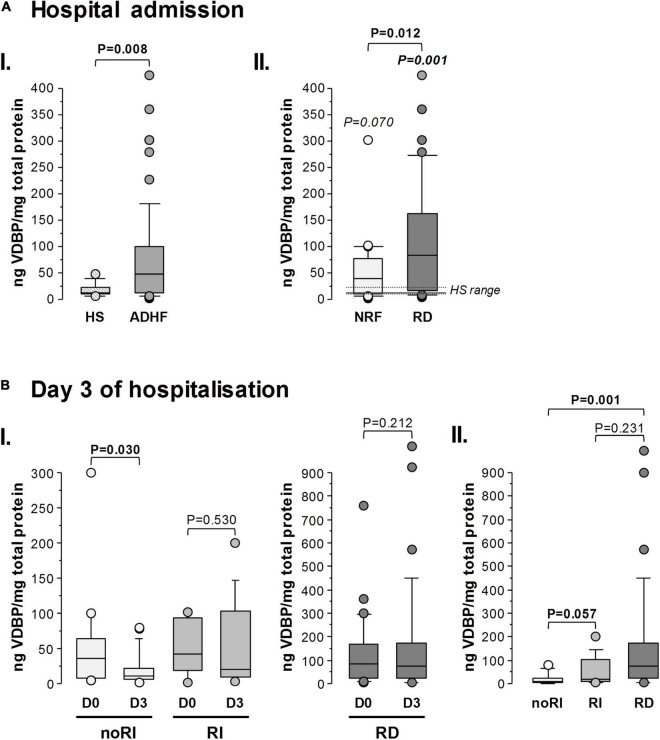
Urinary VDBP in patients with ADHF during hospitalisation: validation studies. Box plot diagrams [median (IQR)] refer to levels of urinary VDBP quantified by ELISA. VDBP in urine was normalised by the total protein content in each sample and expressed as ng VDBP/mg total protein. The Mann-Whitney test was performed for comparison between groups at: **(A)** hospital admission, (I) patients with ADHF (*N* = 64) vs. healthy subjects (HS, *N* = 13), (II) patients with ADHF with glomerular filtration rate (GFR) below (*N* = 34) and above 60 ml/min/1.73 m^2^ at hospital admission (*N* = 30); **(B)** 3 days of hospitalisation, (I) comparison between day 0 (D0) and day 3 (D3) for patients with ADHF maintaining normal renal function (noRI: *N* = 21) and presenting with renal function deterioration (RI: *N* = 13) after 3 days hospitalisation, and in patients with present renal dysfunction at hospitalisation (RD, *N* = 30); and (II) comparison between ADHF subgroups (Mann-Whitney test was performed after the Kruskal-Wallis test for multiple group comparison showed *P*-value < 0.05).

In validation studies (*N* = 64), patients with ADHF presented significantly higher VDBP levels in urine (48.2 [13.0–100.8] ng/mg total protein) than those in the defined healthy range (12.6 [11.2–22.0] ng/mg total protein, *P* < 0.01) when quantified by specific ELISA (Figure 3A). More specifically, the RD and NRF groups had median uVDBP levels (85.5 [17.7–171.7] ng/mg total protein and 38.8 [10.2–76.0] ng/mg total protein, respectively) sixfold (*P* = 0.001) and threefold (*P* = 0.072), respectively, above the median value of the defined healthy range. In addition, patients with ADHF with pathological GFR levels at hospital admission (RD group) had higher VDBP loss in urine than those in the NRF group (*P* = 0.012) (Figure 3A).

Urinary levels of VDBP (uVDBP) in patients with ADHF at hospital admission (day 0) significantly correlated with NT-proBNP (Rho = 0.337, *P* = 0.019) and GFR (Rho = –0.379, *P* = 0.005) in patients with ADHF. In contrast, uVDBP did not associate with LVEF (Rho = 0.013, *P* = 0.924), and no differences were found in uVDBP levels between patients with reduced LVEF (<40%) and preserved LVEF (>50%, *P* = 0.885). Moreover, the uVDBP levels did not differ significantly between patients with and without atrial fibrillation (*P* = 0.337) or previous cardiovascular disease (*P* = 0.437). To notice, levels of uVDBP were not affected by age (Rho = 0.106, *P* = 0.437) or sex (*P* = 0.563) nor by common comorbidities such as hypertension (75%, *P* = 0.0851) and dyslipidaemia (70%, *P* = 0.212, Supplementary Table 3).

Vitamin D binding protein has been previously studied within diabetic nephropathy. The 44% of the patients in the ADHF study population had diabetes, with a trend towards higher frequency of diabetes in the RD compared with that in the NRF group (*P* = 0.079; Table 1). However, uVDBP levels did not significantly differ between diabetic and non-diabetic patients (*P* = 0.635), neither in the NRF group (*P* = 0.572) nor in the RD group (*P* = 0.594, see Supplementary Table 3). Furthermore, as shown in Supplementary Figure 4, patients with diabetes were homogenously distributed when the population was divided in VDBP tertiles. A similar pattern was observed when the groups with normal and reduced renal function at hospital admission were separately considered (NRF group vs. RD group; χ^2^: *P* = 0.789 for differences in the pattern distribution of patients with diabetes). Moreover, as summarised in Supplementary Table 4, urinary VDBP levels did not depend on background medication, including statins, anticoagulants, and antiplatelets, beta blockers, and ACE inhibitors (*P* > 0.05 between treated and non-treated patients for all medications).

### Vitamin D Binding Protein Levels at Day 3 of Hospitalisation

In total, 38.2% of patients in the NRF group presented a worsening of renal function to pathological values between day 5 hospitalisation and hospital discharge (RI group; *N* = 13), whereas renal function was not significantly affected in the remaining 61.8% of the patients in the NRF group (noRI group; *N* = 21), with GFR > 60 ml/min/1.73 m^2^ (see Figure 1).

At day 3, as shown in Figure 3B, patients who did not develop renal injury (noRI group) during the hospitalisation period showed a significant drop of uVDBP to values within the HS range (noRI group: 11.1 [5.7–21.2] ng/mg total protein at day 3; *P* = 0.030 in comparison with values at day 0). In contrast, uVDBP levels remained high and did not significantly differ from values at admission (*P* = 0.530) in those patients who were going to develop renal injury (RI group) before hospital discharge. It is to notice, however, that patients in the RI group still presented GFR levels within the normal physiological range (68.1 [61.5–77.8] ml/min/1.73 m^2^) at day 3 of hospitalisation.

Patients in the RD group (GFR: 42.3 [31.6–52.4] ml/min/1.73 m^2^) maintained elevated levels of uVDBP (85.7 [22.0–173.9] ng/mg total protein) at day 3 with a median value > sixfold higher than in the noRI group (*P* = 0.007; Figure 3B).

**FIGURE 4:**
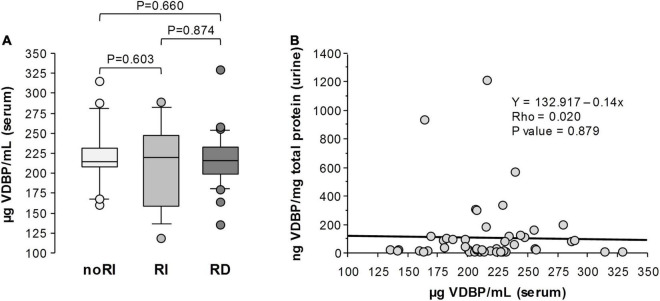
Serum VDBP in patients with ADHF at day 3 of hospitalisation. **(A)** Box plot diagrams [median (IQR)] of VDBP levels quantified by ELISA in serum of patients with ADHF already presenting renal dysfunction at hospital admission (RD), developing renal function deterioration during hospitalisation (RI), and maintaining normal renal function (noRI). Groups’ comparisons were made by the Kruskal-Wallis and Mann-Whitney test. **(B)** Spearman correlation (Rho) between VDBP levels in serum and urine in patients with ADHF at day 3 of hospitalisation.

The analysis of sera samples obtained at day 3 of hospitalisation did not show significant differences in VDBP levels between noRI and RI groups (Figure 4A; *P* = 0.603). Additionally, RD group did not present different levels with noRI patients (*P* = 0.660, Figure 4A) nor with RI patients (*P* = 0.874, Figure 4A). No correlation was found between urine and serum VDBP levels in patients with ADHF (Rho = 0.020; *P* = 0.879; Figure 4B). In addition, at day 3, no correlation was shown between GFR and serum VDBP levels (Rho = 0.060, *P* = 0.657) in ADHF, whereas GFR significantly correlated with VDBP levels in urine (Rho = –0.472, *P* = 0.001) (Supplementary Figure 3).

### Urinary Vitamin D Binding Protein Levels Added to Cystatin C and KIM-1 Improved Discrimination of Patients With Acute Decompensated Heart Failure With Renal Deterioration During Hospitalisation

**FIGURE 5:**
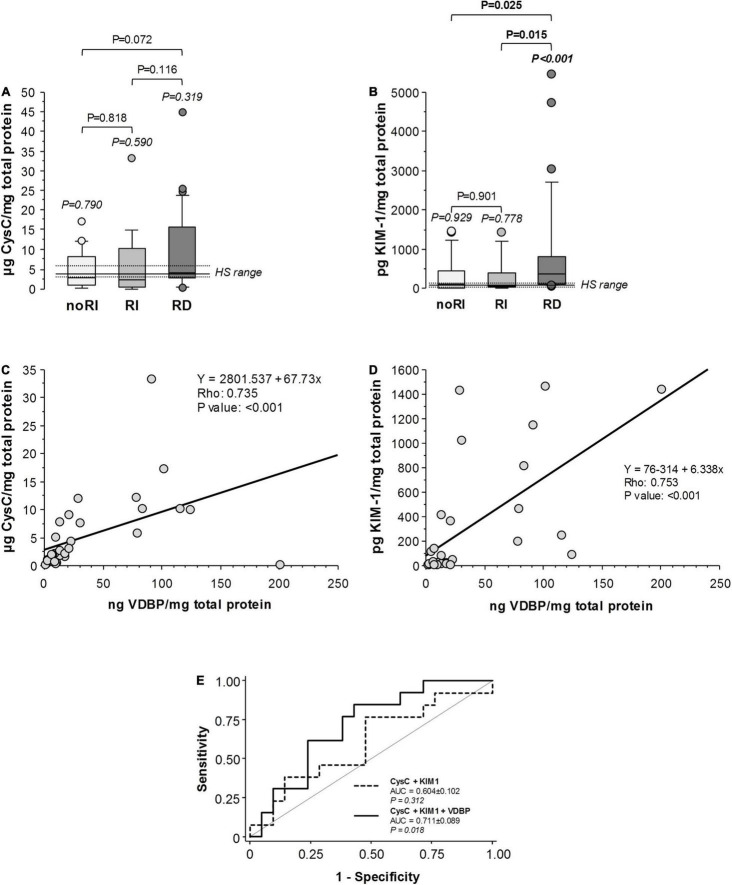
Markers of tubular damage (urinary CysC and KIM-1) in patients with ADHF at day 3 of hospitalisation. Box plot diagrams [median (IQR)] refer to **(A)** cystatin C (CysC) and **(B)** Kidney Injury Molecule 1 (KIM-1) in the subgroups of patients with ADHF as described in Figure 4A. **(C,D)** Spearman correlation (Rho) between urinary VDBP levels and CysC and KIM-1, respectively, in all patients with ADHF. **(E)** ROC curve analyses (C-Statistics) for the prediction of renal function deterioration presentation in patients with ADHF (*N* = 34). AUC indicates area under the curve.

Cystatin C and KIM-1 have been proposed to change during early renal injury (10, 11, 30). Cystatin C in urine of patients with ADHF negatively correlated with GFR at admission (Rho = –0.363; *P* = 0.007), and values were twofold higher than those in the HS group (6.07 [2.34–11.04] vs. 3.90 [3.12–5.72] μg/mg total protein), although differences did not achieve statistical significance (*P* = 0.189). At day 3 of hospitalisation, uCysC did not differ between ADHF groups, although an increasing trend was observed in RD patients when compared with those maintaining normal renal function (noRI) during the hospitalisation period (*P* = 0.072) (Figure 5A). Similarly, the highest KIM-1 levels in urine (uKIM-1) at day 3 were found in the RD group with significant differences when compared with the RI and noRI groups (Figure 5B). However, no differences were observed between noRI and RI groups at 3 days of hospitalisation (Figure 5B).

Levels of uVDBP negatively correlated with GFR, and a significant positive correlation was found with uCysC and uKIM-1 in patients with ADHF at day 3 of hospitalisation (Supplementary Table 5). Stronger positive correlations levels were found when only patients with ADHF with GFR above 60 ml/min/1.73 m^2^ were considered (noRI and RI groups; Figures 5C,D).

We tested whether each one of the proteins (uVDBP, uCysC, and uKIM-1) had the potential to discriminate renal injury in patients with ADHF by the ROC curve analysis (Supplementary Table 6A). None of the 3 proteins presented a significant discriminating ability when taken individually, with urinary levels of VDBP presenting the largest area under the curve (AUC = 0.579) compared with those of KIM-1 (AUC = 0.487) and cystatin C (AUC = 0.476), although differences among them did not achieve statistical significance (Supplementary Table 6B). The combination of cystatin C and KIM-1 showed a greater area under the curve but still not statistically significant (AUC = 0.604, *P* = 0.312). However, the addition of VDBP levels to this combination significantly increased the discriminating value for renal function deterioration in patients with ADHF (AUC = 0.711, *P* = 0.018, Figure 5E), whereas the combination of VDBP with either cystatin C or KIM-1 alone was not significant (*P* = 0.428 and *P* = 0.089, respectively).

Due to the impact of age in renal dysfunction, the combination of aggregating VDBP, CysC, and KIM-1 for an early discrimination of patients with ADHF with subclinical renal function deterioration during hospitalisation was further reanalysed in an age-adjusted subgroup of patients (age; RI vs. noRI subgroups: 74 [69–77] vs. 69 [63–72] years, *P* = 0.304; *N* = 13/group). As shown in Supplementary Figure 5, in this age-adjusted cohort, the discrimination power of combining VDBP, CysC, and KIM-1 remained statistic significantly (AUC: 0.711 [0.536–0.885], *P* = 0.018). Adding age to the 3-variable cluster did not significantly modify the AUC to early discriminate patients with and without deterioration of kidney function during hospitalisation.

## Discussion

Heart and kidneys are closely interconnected through complex bidirectional mechanistic interactions in their underlying pathophysiology (31), which is collectively known as cardiorenal syndrome (6) and often referred to as type-1 cardiorenal syndrome when having a primary and acute cardiac condition, such as ADHF, that triggers acute kidney injury (4). In patients hospitalised with ADHF, occurrence of acute kidney injury during the treatment frequently affects key therapeutic decisions, resulting in incomplete decongestion of the patients (5).

There is a growing consensus about the poor effectiveness of serum creatinine measurement to detect initial stages of cardiorenal syndrome type 1 (32) and the urgent need of more reliable biological variables for early detection of kidney function deterioration in patients with ADHF (2, 4–6) in order to better stratify their risk during the first days of hospitalisation. NGAL (33), KIM-1 (34), and cystatin C (35) had provided promising results at detecting severe renal damage in patients with ADHF and other cardiac syndromes; however, negative studies (15, 32, 36–38) have also been reported. There is certainly controversy regarding their relative diagnostic power.

Therefore, we performed an exploratory proteomic study aimed to identify urinary protein associated with early changes of renal function impairment in patients hospitalised with diagnostic of ADHF. Urinary proteins have been extensively studied in acute heart failure as they may be more sensitive to renal injury and structural damage than serum creatinine. Till present, however, there is insufficient evidence to translate the usefulness of measuring these proteins as the clinical diagnostic tool.

Our hypothesis-generating study is based on an untargeted mass-spectrometry approach. We identified a differential pattern of proteins involved in vitamin A and D homeostasis in prospectively collected spot-urine samples of patients with ADHF when arriving to the emergency room. More specifically, we have found that urinary VDBP levels are markedly elevated in patients with ADHF, being changes more evident in those patients with reduced GFR, suggesting an association between low GFR and high VDBP levels. Unfortunately, we cannot discern if low GFR levels in the RD patients evidenced a chronic renal dysfunction or were a result of the acute decompensated event. Our findings are consistent with previous reports on urinary VDBP in the setting of pathologies associated to nephropathies in humans. In systemic lupus erythematosus, urinary VDBP was significantly elevated in patients with lupus nephritis and associated with disease severity (39). In addition, VDBP has been studied in diabetic nephropathy and has shown to correlate with microalbuminuria, suggesting a diagnostic value in this pathology (40). Interestingly, it was previously reported that urinary VDBP was strongly and consistently elevated in rats with induced nephropathy very early in the course of the disease (25). VDBP has also been associated with major contrast-induced nephropathy events 90 days after exposure to contrast media in patients undergoing coronary angioplasty (24). To our knowledge, however, it was not shown so far that the changes in urinary VDBP levels are detected before renal injury is evidenced in hospitalised patients with ADHF.

It is to notice that urinary VDBP significantly correlated with plasma NT-proBNP and the level of GFR, suggesting VDBP as a protein associated with cardiorenal syndrome. This finding is in line with a previous study using NT-proBNP and cystatin C, as a marker of renal function, to identify the cardiorenal status in patients with refractory heat failure on cardiac resynchronisation therapy (41). The fact that no relationship between VDBP and ejection fraction was found might result from the heterogeneity of our study population. Indeed, patients with heart failure with reduced and preserved ejection fraction are frequently considered as two different phenotype entities with differences in pathophysiological pathways deriving in the cardiorenal syndrome (42, 43).

As suggested before, high urinary VDBP in patients with ADHF at hospital admission may partially reflect a temporary kidney function deterioration event due to impaired renal perfusion as consequence of low cardiac output and systemic venous congestion (8, 44). However, the maintained urinary VDBP loss in patients with ADHF with GFR below 60 ml/min/1.73 m^2^ after 3 days of hospitalisation suggest that high VDBP levels in urine associate to a renal structural damage in these patients, that might derive into cardiorenal syndrome type 1.

Indeed, among patients with ADHF with normal renal function at hospital admission (NRF group), urinary VDBP remains high at day 3 in those patients presenting worsening of the renal function at a later stage of the hospitalisation period, while patients who maintained normal renal function presented a decrease of urinary VDBP into the healthy range. Interestingly, these changes at day 3 were observed before any apparent pathological change in plasma creatinine levels and the GFR, that when occurred it was detected after 4–5 days hospitalisation. This finding suggests that urinary VDBP is sensitive to early pathological changes occurring in kidney and identifying those patients with subclinical kidney function deterioration.

High urinary VDBP levels in patients with ADHF may result from renal tubular injury or reflect hemodynamic or functional changes in GFR (24, 25). Our results showed no association between urinary VDBP loss and serum levels of VDBP supporting a tubular damage leading to diminished reabsorption and consequently higher urinary excretion as the most possible process accounting for the high VDBP levels in urine of patients with ADHF. In this respect, Chaykovska et al. (24) also proposed the increase of VDBP levels could be associated with the severity of renal damage.

Although the underlying mechanisms remain unclear, we could speculate that elevated levels of VDBP in urine could regulate tubular cell function after endocytosis *via* megalin, since megalin is involved in the active uptake of VDBP complexed to 25-hydroxy vitamin D3 in the proximal tubules (45). A reduced uptake of VDBP, as in kidney disease conditions, could lead to intrarenal losses of VDBP and vitamin D metabolites (45). Further studies are needed to evidence whether VDBP is a relevant cardiorenal connector protein transmitting heart-to-kidney signals.

Other proteins in urine including KIM-1 and cystatin C have been related to renal failure and more specifically to tubular damage (14, 15). Supporting this finding, at day 3 of hospitalisation, KIM-1 levels were significantly elevated only in those patients with a pathological GFR. Also, a trend to higher values was observed in the levels of uCysC although it did not achieve statistical significance. In contrast, patients with ADHF with GFR > 60 ml/min/1.73 m^2^ at day 3 of hospitalisation had KIM-1 and cystatin C levels that did not differ from the normal healthy range regardless of the subsequent evolution of the kidney function. Different pattern of VDBP and cystatin C and KIM-1 in urine of patients with ADHF at day 3 could be due to differences in the time response of the cellular processes occurring at the proximal tubular cells after injury. Thus, KIM-1 is a protein expressed in proximal tubular cells after injury, but barely found in normal kidney (46), whereas the presence of cystatin C and VDBP in urine result of a deficient endocytic receptor-mediated uptake, catabolism, and degradation of this protein by the tubular cells (47, 48). Further studies are guaranteed to better understand whether differences in the endocytic process and/or cell catabolism account for the VDBP and cystatin C patterns in urine of patients with ADHF.

The positive correlation of urinary VDBP with cystatin C and KIM-1 and day 3 of hospitalisation strongly suggest that renal tubular insult accounts for acute kidney function deterioration in patients with ADHF. Differences with two previous studies suggesting a lack of association between worsening of renal function and tubular injury in ADHF might be explained by the fact that these studies just focused on the expression/secretion of proteins related to tubular cell injury such as KIM-1 and NGAL (49, 50), without considering other molecules reflecting a dysfunctional cell phenotype.

Subsequently, the ROC analysis was carried out to study the value of VDBP compared with cystatin C and KIM-1 in association with incident kidney function deterioration in patients hospitalised with ADHF. In our study population, none of the three proteins showed enough discriminative power when considered separately, nor did it when considering the joint detection of cystatin C and KIM-1. However, when VDBP was added the AUC achieved statistical significance, suggesting that urine VDBP combined with urine values of cystatin C and KIM-1 might serve as new diagnostic tool to discriminate ADHF patients regarding their risk of developing renal function deterioration during hospitalisation.

Age is an important factor of kidney injury risk and development (51). In this respect, we want to highlight as a study limitation the age difference between ADHF patients with and without renal dysfunction at admission. However, VDBP levels did not correlate with age in our study population, suggesting the independence of both variables. In addition, it is interesting to notice that the discriminating power of VDBP, clustered with cystatin C and KIM-1 for kidney function deterioration in patients hospitalised with ADHF is maintained when age-adjusted ADHF groups were compared. Thus, to the best of our knowledge, this is the first study reporting an association between urinary VDBP and renal worsening in ADHF during hospitalisation. Still, the limited sample size and high heterogeneity of the study population in their characteristics, etiopathology, and disease background need to be recognised. Therefore, further studies in larger groups are needed to confirm our findings and validate the impact of urinary VDBP in ADHF and incident kidney disease.

Vitamin D binding protein is forming a complex with the 25-(OH) vitamin D3 when present in urine. Tubular reabsorption of this complex is a critical process into the conversion of vitamin D into its active metabolite that is secreted into the circulation (52). Damage to proximal tubules in ADHF leading to a deficient reabsorption of VDBP-25-(OH) vitamin D3 complex could result in diminished amounts of vitamin D active metabolite, also known as 1,25-dihydroxycholecalciferol and calcitriol. In this respect, vitamin D deficiency has been previously associated to several diseases concerning renal failure (53, 54), cardiovascular health (55), certain types of cancer, type 2 diabetes, metabolic syndrome, inflammatory bowel disease, and several others (56, 57). Vitamin D has been studied within chronic heart failure (58); however, to our knowledge, there are no publications associating ADHF with vitamin D.

In summary, VDBP has been consistently detected by 2DE-MS/MS as two spots in urine of patients with ADHF at hospital admission in this exploratory study, wherein the patients with ADHF presented higher urinary levels than healthy subjects. Additionally, patients with ADHF presenting kidney dysfunction at hospitalisation showed the highest urinary loss of VDBP. A significant drop in urinary VDBP is observed within 3 days in patients with normal renal function during the hospitalisation period. On the contrary, those patients who developed renal function deterioration maintained high levels of VDBP loss in urine, while their GFR levels were within normal physiological range at that time. When performing ROC analysis, the addition of urinary VDBP to renal injury markers such as uCysC and KIM-1 gave statistical power to anticipate renal damage. Therefore, a daily follow-up of VDBP urinary secretion could help to determine whether patients’ kidneys are prone to failure and prevent it using more appropriate and personalised therapeutic techniques.

## Data Availability Statement

The original contributions presented in the study are included in the article/Supplementary Material, further inquiries can be directed to the corresponding author.

## Ethics Statement

The studies involving human participants were reviewed and approved by the Ethical Committee for Clinical Research from Hospital Santa Cruz y San Pablo in March 2016 (ref number 16/022). The patients/participants provided their written informed consent to participate in this study.

## Author Contributions

XG-M, LB, and TP: study concept and design. LL and X-GM: patient inclusion, clinical data, and sample acquisition. ED-R, MG-A, and TP: methodology and formal analysis. ED-R, LB, and TP: statistical analysis and result interpretation. ED-R, LB, and TP: writing–original draft preparation. ED-R, MG-A, LL, XG-M, LB, and TP: manuscript review and editing. LB and TP: funding acquisition. All authors have read and agreed to the submitted version of the manuscript.

## Conflict of Interest

LB received institutional research grants from AstraZeneca; consultancy fees from Sanofi, Pfizer and Novartis; speaker fees from Lilly, Pfizer, and AstraZeneca. TP and LB are shareholders of the academic spin-off companies GlyCardial Diagnostics S.L. and Ivestatin Therapeutics S.L. All unrelated to the present work. The remaining authors declare that the research was conducted in the absence of any commercial or financial relationships that could be construed as a potential conflict of interest.

## Publisher’s Note

All claims expressed in this article are solely those of the authors and do not necessarily represent those of their affiliated organizations, or those of the publisher, the editors and the reviewers. Any product that may be evaluated in this article, or claim that may be made by its manufacturer, is not guaranteed or endorsed by the publisher.

## Abbreviations

ADHF: acute decompensated heart failure
CysC: cystatin C
GFR: glomerular filtration rate
KIM-1: kidney injury molecule 1
LVEF: left ventricular ejection fraction
NGAL: neutrophil gelatinase-associated lipocalin
RD: renal dysfunction patients art hospitalisation
noRI: patients who did not develop renal injury
NRF: normal renal function patients at hospitalisation
RI: patients who developed renal injury
VDBP: vitamin D binding protein
2DE: two-dimension electrophoresis.
