# Supplementary material for: Vitamin D Binding Protein and Renal Injury in Acute Decompensated Heart Failure
Source: Front Cardiovasc Med. 2022 Jun 9;9:829490. doi: 10.3389/fcvm.2022.829490 (PMC9222131; doi:10.3389/fcvm.2022.829490)
Supplement: Supplementary file 1 [file Data_Sheet_1.PDF]

Supplementary material

Vitamin D Binding Protein and renal injury in acute decompensated heart failure

Elisa Diaz-Riera[1,2]; Maísa García-Arguinzonis[1], Laura López[3], Xavier Garcia-Moll[3,4], Lina Badimon L[1,4,5], Padró, T[1,4*]

[1]Cardiovascular-Program ICCC; Research Institute – Hospital Santa Creu i Sant Pau, IIB-Sant Pau, Barcelona, Spain

[2]Faculty of Medicine, University of Barcelona (UB), 08036 Barcelona, Spain

[3]Cardiology Department, Hospital Santa Creu i Sant Pau, Barcelona, Spain

[4]Centro de Investigación Biomédica en Red Cardiovascular (CIBERCV) Instituto de Salud Carlos III, Madrid, Spain; [5]Cardiovascular Research Chair, UAB, Barcelona, Spain

***Correspondence:**
Dr Teresa Padro
tpadro@santpau.cat

Supplementary Table I: ADHF patients' characteristics in the discovery phase at hospital admission by 2 dimension electrophoresis

	All patients N=17	ADHF-NRF n=12	ADHF-RD n=5	P-value
**DEMOGRAPHIC CHARACTERISTICS; median [IQR]**				
Female/male, N	3/14	2/10	1/4	
Age, years	72.0 [69.0-76.0]	71.0 [63.5-76.5]	74.0 [71.0-75.0]	0.561
Weight, kg	75.8 [65.8-86.6]	82.1 [65.8-93.0]	73.0 [61.3-78.7]	0.322
**KIDNEY FUNCTION MARKERS**				
Creatinine, µmol/L	101 [73-131]	86 [66.5-103]	191 [147-197]	**0.002**
Glomerular filtration (MDRD-4)	68 [43.2-81.7]	73.6 [65.3-109.5]	32.2 [30.7-35.2]	**0.002**
Urea, mmol/L	9.0 [5.8-13.1]	6.5 [5.3-9.1]	23.1 [22.1-25.5]	**0.002**
**CARDIAC FUNCTION MARKERS**				
NT-proBNP, ng/L	2424 [1734-4570]	2094 [1346-4293]	2591 [2256-19677]	0.157
Left ventricular ejection fraction (LVEF), %	48.0 [33.0-56.0]	47.5 [34.0-58.0]	51.0 [33.0-55.0]	0.832
Preserved LVEF, N (%)	8 (47)	5 (42)	3 (60)	
Reduced LVEF, N (%)	6 (35)	4 (33)	2 (40)	0.462
Mid-range LVEF, N (%)	3 (18)	3 (25)	0 (0)	
Atrial fibrillation, N (%)	11 (65)	7 (58)	4 (80)	0.600
Cardiovascular disease, N (%)	4 (24)	2 (17)	2 (40)	0.538
**OTHER BIOCHEMICAL MARKERS**				
Haemoglobin, g/L	122 [106-139]	126 [114-141.5]	106 [104-121]	0.225
**RISK FACTORS; N (%)**				
Active smoking	3 (18)	3 (25)	0 (0)	>0.999
Hypertension	13 (77)	9 (75)	4 (80)	>0.999
Pulmonary hypertension	6 (35)	4 (33)	2 (40)	>0.999
Diabetes mellitus type 2	6 (35)	3 (25)	3 (60)	0.280
Dyslipidaemia	12 (71)	9 (67)	3 (60)	0.600
**BACKGROUND MEDICATION; N (%)**				
Diuretics	10 (59)	5 (42)	5 (100)	**0.044**
Statins	9 (53)	6 (35)	3 (60)	>0.999
Anticoagulants	9 (53)	5 (42)	4 (80)	0.294
Antiplatelet agents	7 (41)	5 (42)	2 (40)	>0.999
Beta-blockers	11 (65)	6 (50)	5 (100)	0.102
Antiarrhythmic agents	0 (0)	0 (0)	0 (0)	>0.999
Antidiabetics	4 (24)	1 (8)	3 (60)	0.053
Insulin	3 (18)	0 (0)	3 (60)	**0.015**
Oral antidiabetic agents	3 (18)	1 (8)	2 (40)	0.191
ACE inhibitor/ARB	13 (76)	9 (75)	4 (80)	>0.999

P values calculated with Fisher exact test, except for LVEF where χ^2 was used. Diuretics: hydrochlorothiazide, furosemide, eplerenone, and spironolactone. Statins: atorvastatin, pravastatin, simvastatin, ezetimibe, fenofibrates. Anticoagulants: warfarin, acenocumarol, bemiparin, heparin, tinzaparin, dabigatran, rivaroxaban, edoxaban, and apixaban. Antiplatelet agents: acetylsalicylic acid and clopidogrel. Beta-blockers: bisoprolol and carvedilol. Antiarrhythmic agents: amiodarone. Oral antidiabetic agents: metformin and repaglinide. Angiotensin-converting enzyme inhibitors (ACEI) include: captopril, enalapril, and ramipril. Angiotensin receptor blockers (ARB): losartan, olmesartan, and valsartan.

Supplementary Table II: Proteins associated to vitamin metabolism presenting a differential detection pattern in urine of ADFH at hospital admission.

Protein name	Gene name	Swiss Prot number	Theoretical pI	Molar weight (KDa)	MASCOT Score	Function
Retinol-binding protein 4	*RBP4*	P02753	5.27	23.0	66*	Vitamin A
Transthyretin	*TTR*	P02766	5.31	15.9	61*	
Vitamin D-binding protein	*GC*	P02774	5.22	21.5	72*	Vitamin D

Proteins identified by 2DE-MS/MS. MASCOT score refers to detection by MS/MS.

Supplementary Table III: Urine VDBP levels obtained by immunoassay according to patients' characteristics and risk factors at hospital admission.

	ADHF patients		ADHF-NRF		ADHF-RD	
	N	Median [IQR]	N	Median [IQR]	N	Median [IQR]
**Sex**						
Male	44	49.2 [12.3-125.7]	25	35.4 [12.0-78.0]	19	125.7 [32.6-227.5]
Female	20	47.2 [13.6-85.5]	9	41.6 [8.0-76.0]	11	54.5 [17.7-118.4]
P value		*0.563*		*0.823*		*0.209*
**LVEF**						
Reduced (<40%)	27	40.0 [20.5-97.3]	18	38.8 [20.8-71.6]	9	125.7 [12.0-161.2]
Mid-range (40-49%)	10	63.4 [26.9-141.3]	5	43.7 [23.4-74.0]	5	130.8 [46.7-270.6]
Preserved (>50%)	27	64.7 [10.3-108.4]	11	10.3[5.3-78.0]	16	76.6 [42.2-163.3]
P value		*0.815*		*0.598*		*0.773*
**Atrial fibrillation**						
No	32	43.6 [10.3-98.3]	17	30.2 [8.6-45.8]	15	91.7 [32.6-161.2]
Yes	31	71.6 [17.7-102.2]	16	71.6 [12.0-95.1]	15	73.6 [17.7-171.7]
P value		*0.337*		*0.123*		*0.961*
**Cardiovascular disease**						
No	44	46.4 [11.1-110.3]	24	30.2 [8.1-71.6]	20	91.7 [17.7-163.3]
Yes	20	55.5 [30.0-94.6]	10	45.4 [33.1-86.0]	10	64.7 [22.3-236.6]
P value		*0.538*		*0.205*		*0.915*
**Arterial hypertension**						
No	16	33.1 [10.3-57.1]	12	33.1 [8.6-45.7]	4	52.7 [12.8-120.7]
Yes	47	69.6 [20.2-125.7]	21	12.0 [41.6-94.0]	26	85.5 [32.6-180.5]
P value		*0.085*		*0.383*		*0.375*
**Dyslipidaemia**						
No	19	30.2 [8.6-91-7]	13	28.8 [8.6-45.8]	6	91.7 [13.6-279.1]
Yes	44	61.8 [20.2-118.4]	20	41.6 [12.0-94.0]	24	83.3 [32.6-163.3]
P value		*0.212*		*0.313*		*0.852*
**Diabetes mellitus type II**						
No	35	48.1 [12.0-99.4]	22	35.7 [8.3-67.6]	13	118.4 [47.1-161.2]
Yes	28	61.8 [20.2-171.7]	11	38.8 [20.8-76.0]	17	64.7 [12.3-180.5]
P value		*0.635*		*0.572*		*0.594*
**Pulmonary hypertension**						
No	48	49.2 [17.7-118.4]	26	40.0 [12.0-94.0]	22	85.5 [32.6-163.3]
Yes	16	45.8 [8.6-76.0]	8	27.4 [5.4-71.6]	8	76.7 [12.0-227.5]
P value		*0.388*		*0.359*		*0.816*

LVEF: left ventricular ejection fraction. Values given in ng VDBP/mg total protein.

Supplementary Table IV: Effect of background medication on urine VDBP levels in ADHF patients at hospital admission

	ADHF patients		ADHF-NRF		ADHF-RD	
	N	Median [IQR]	N	Median [IQR]	N	Median [IQR]
**Diuretics**						
No	16	41.6 [8.6-81.1]	13	30.2 [8.1-78.0]	3	220.9 [83.1-360.7]
Yes	48	49.2 [13.6-118.4]	21	40.0 [12.0-76.0]	27	85.5 [17.7-163.3]
P value		*0.567*		*0.822*		*0.309*
**Statins**						
No	23	38.0 [10.3-81.1]	16	25.2 [8.3-64.4]	7	96.4 [17.7-171.7]
Yes	41	55.5 [20.8-125.7]	18	41.1 [20.8-94.0]	23	85.5 [32.6-163.3]
P value		*0.213*		*0.288*		*0.907*
**Anticoagulants**						
No	38	45.7 [11.3-95.0]	20	34.5 [9.5-64.4]	18	74.4 [22.4-143.3]
Yes	26	62.6 [15.7-156.5]	14	41.1 [12.0-94.0]	12	149.7 [17.7-279.1]
P value		*0.354*		*0.630*		*0.236*
**Antiplatelet agents**						
No	28	44.7 [11.1-120.7]	15	11.1 [6.0-64.4]	13	88.6 [29.9-162.2]
Yes	36	55.5 [24.1-100.8]	19	41.6 [27.4-94.0]	17	81.1 [12.3-180.5]
P value		*0.477*		*0.121*		*0.961*
**Beta-blockers**						
No	20	47.1 [10.3-102.2]	13	12.0 [5.4-76.0]	7	122.0 [47.1-161.2]
Yes	44	49.2 [17.7-99.4]	21	41.3 [20.8-78.0]	23	81.1 [13.6-180.5]
P value		*0.527*		*0.281*		*0.683*
**Antiarrhythmic agents**						
No	58	44.4 [12.0-102.2]	30	30.2 [8.6-71.6]	28	85.5 [32.6-171.7]
Yes	6	62.6 [31.9-78.0]	4	62.6 [40.6-77.0]	2	83.7 [17.7-149.7]
P value		*0.791*		*0.255*		*0.781*
**Antidiabetic agents**						
No	39	46.5 [11.1-98.9]	26	30.2 [8.1-78.0]	13	118.4 [47.1-161.2]
Yes	25	64.7 [26.4-176.1]	8	55.2 [31.9-76.0]	17	64.7 [12.3-180.5]
P value		*0.330*		*0.236*		*0.594*
**ACE inhibitor/ARB**						
No	21	61.8 [13.6-161.2]	8	45.6 [8.6-78.0]	13	96.7 [29.9-176.1]
Yes	43	45.8 [12.3-95.1]	26	35.4 [12.0-76.0]	17	85.5 [12.3-163.3]
P value		*0.452*		*>0.999*		*0.626*

P values calculated using Mann-Whitney. Median values given in ng VDBP/mg total protein. Diuretics: hydrochlorothiazide, furosemide, eplerenone, and spironolactone. Statins: atorvastatin, pravastatin, simvastatin, ezetimibe, fenofibrates. Anticoagulants: warfarin, acenocumarol, bemiparin, heparin, tinzaparin, dabigatran, rivaroxaban, edoxaban, and apixaban. Antiplatelet agents: acetylsalicylic acid and clopidogrel. Beta-blockers: bisoprolol and carvedilol. Antiarrhythmic agents: amiodarone. Oral antidiabetic agents: metformin and repaglinide. Angiotensin-converting enzyme inhibitors (ACEI) include: captopril, enalapril, and ramipril. Angiotensin receptor blockers (ARB): losartan, olmesartan, and valsartan.

Supplementary Table V: Correlation values between VDBP and markers of renal function (GFR, Cystatin C) and kidney damage (KIM-1) in ADHF patients (N=64) at day 3 of hospitalisation.

	VDBP			
	**Correlation**	**Lower limit**	**Upper limit**	**P value**
**GFR**	-0.396	-0.602	-0.141	***0.003***
**Cystatin C**	0.596	0.389	0.746	***<0.001***
**KIM-1**	0.477	0.237	0.662	***<0.001***

VDBP: Vitamin D Binding Protein; GFR: glomerular filtration rate; KIM-1: Kidney Injury Molecule 1

Supplementary Table VI: ROC (associated receiver operating characteristic) curve (AUC) analysis for determining discriminatory power of urine VDBP for incident AKI in ADHF patients at day 3 of hospitalisation.

A. ROC curves of urinary levels of VDBP, cystatin C, KIM-1, and the combination of all three proteins.

**Protein**	**AUC Area**	**Error deviation**	**Lower limit**	**Upper limit**	**P-value**
VDBP	0.579	0.105	0.374	0.783	*0.446*
CysC	0.476	0.105	0.270	0.682	*0.818*
KIM-1	0.487	0.103	0.284	0.690	*0.901*
CysC + KIM-1 + **VDBP**	0.711	0.089	0.536	0.885	***0.018***

VDBP: Vitamin D Binding Protein; CysC: Cystatin C; KIM1: Kidney Injury Molecule 1.

B. P values of the comparison of AUC for all three proteins and their combination.

	**VDBP**	CysC	KIM-1	CysC + KIM-1 + **VDBP**
**VDBP**		0.479	0.532	0.338
CysC	0.479		0.940	0.088
KIM-1	0.532	0.940		0.100
CysC + KIM-1 + **VDBP**	0.338	0.088	0.100	

VDBP: Vitamin D Binding Protein; CysC: Cystatin C; KIM1: Kidney Injury Molecule 1.

Supplementary Table VII: Urine Cystatin C levels according to patients' characteristics and risk factors at hospital admission.

		**ADHF patients**		**ADHF-NRF**		**ADHF-RD**
	N	Median [IQR]	N	Median [IQR]	N	Median [IQR]
**Sex**						
Male	44	3.97 [2.10-11.44]	25	3.12 [2.02-8.77]	19	9.03 [2.39-14.41]
Female	20	6.32 [2.74-9.84]	9	4.97 [2.08-8.86]	11	9.21 [4.85-20.75]
P value		*0.438*		*0.760*		*0.763*
**LVEF**						
Reduced (<40%)	27	5.86 [2.37-10.02]	18	3.97 [2.34-9.29]	9	9.03 [2.39-13.24]
Mid-range (40-49%)	10	4.33 [2.43-11.64]	5	2.43 [0.79-6.12]	5	9.79 [4.33-22.23]
Preserved (>50%)	27	5.52 [2.04-13.16]	11	3.38 [2.00-6.06]	16	9.21 [2.31-20.75]
P value		*0.958*		*0.327*		*0.862*
**Atrial fibrillation**						
No	32	4.33 [2.10-11.44]	17	2.73 [2.02-8.24]	15	9.03 [3.02-14.88]
Yes	31	4.97 [2.31-10.09]	16	3.97 [2.11-8.77]	15	9.21 [2.31-20.75]
P value		*0.973*		*0.629*		*0.771*
**Cardiovascular disease**						
No	44	4.75 [2.09-9.44]	24	2.73 [2.00-4.97]	20	9.03 [3.49-14.88]
Yes	20	9.56 [2.62-17.35]	10	9.69 [5.86-12.59]	10	6.43 [2.9-27.65]
P value		*0.099*		***0.003***		*>0.999*
**Arterial hypertension**						
No	16	5.46 [2.02-9.29]	12	4.73 [2.02-8.77]	4	7.40 [3.32-10.69]
Yes	47	5.07 [2.22-13.24]	21	2.85 [2.08-8.86]	26	9.03 [2.39-21.12]
P value		*0.532*		*0.819*		*0.539*
**Dyslipidaemia**						
No	19	8.24 [2.11-13.24]	13	7.15 [2.02-9.29]	6	11.44 [4.85-13.24]
Yes	44	4.66 [2.22-9.95]	20	2.85 [2.08-7.74]	24	8.81 [2.39-20.75]
P value		*0.535*		*0.409*		*0.618*
**Diabetes mellitus type II**						
No	35	7.74 [2.22-9.95]	22	3.06 [1.93-8.81]	13	9.95 [6.32-14.88]
Yes	28	3.97 [2.10-13.26]	11	3.97 [2.73-4.97]	17	4.09 [1.79-20.75]
P value		*0.696*		*0.540*		*0.207*
**Pulmonary hypertension**						
No	48	6.32 [2.22-13.26]	26	3.41 [2.02-8.86]	22	9.03 [3.49-21.12]
Yes	16	3.39 [2.11-9.29]	8	3.39 [2.11-6.06]	8	6.07 [1.79-11.44]
P value		*0.233*		*0.760*		*0.180*

LVEF: left ventricular ejection fraction. Median values given in μg CysC/mg total protein.

Supplementary Table VIII: Urine KIM-1 levels according to patients' characteristics and risk factors at day 3 of hospitalisation.

	ADHF patients		ADHF-NRF		ADHF-RD	
	N	Median [IQR]	N	Median [IQR]	N	Median [IQR]
**Sex**						
Male	44	181.0 [23.5-462.6]	25	53.6 [11.3-366.7]	19	383.5 [169.4-862.6]
Female	20	260.1 [41.3-897.2]	9	203.0 [17.4-1022.7]	11	352.4 [81.6-803.4]
P value		*0.806*		*0.598*		*0.509*
**LVEF**						
Reduced (<40%)	27	203.0 [29.4-803.4]	18	89.2 [11.3-366.7]	9	359.8 [121.8-803.4]
Mid-range (40-49%)	10	233.5 [81.6-467.2]	5	467.2 [21.3-1149.9]	5	232.7 [99.5-234.2]
Preserved (>50%)	27	216.9 [20.7-745.6]	11	20.7 [4.9-145.2]	16	568.7 [216.9-907.3]
P value		*0.932*		*0.334*		*0.364*
**Atrial fibrillation**						
No	32	259.4 [84.3-816.6]	17	203.0 [7.4-815.3]	15	408.8 [177.3-862.6]
Yes	31	114.0 [21.3-438.8]	16	39.1 [18.2-195.9]	15	352.4 [82.9-745.6]
P value		*0.260*		*0.589*		*0.343*
**Cardiovascular disease**						
No	44	133.5 [19.8-727.8]	24	23.0 [7.2-341.9]	20	328.0 [110.7-810.6]
Yes	20	322.2 [86.3-657.1]	10	224.8 [53.6-420.7]	10	438.8 [91.0-907.4]
P value		0.253		*0.174*		*0.710*
**Arterial hypertension**						
No	16	129.6 [9.3-416.9]	12	71.7 [7.2-291.7]	4	560.7 [186.3-1935.7]
Yes	47	234.2 [53.1-803.4]	21	53.6 [18.9-815.3]	26	359.8 [99.5-803.4]
P value		*0.352*		*0.653*		*0.724*
**Dyslipidaemia**						
No	19	81.6 [4.4-366.7]	13	18.9 [4.4-216.7]	6	294.0 [81.6-407.3]
Yes	44	240.4 [76.1-1006.8]	20	89.2 [21.0-1045.3]	24	438.8 [121.8-907.4]
P value		***0.009***		*0.060*		*0.250*
**Diabetes mellitus type II**						
No	35	246.6 [18.9-817.8]	22	87.3 [7.4-815.3]	13	407.3 [99.5-817.8]
Yes	28	169.4 [75.4-448.3]	11	53.6 [17.4-114.0]	17	356.1 [121.8-745.6]
P value		*0.945*		*0.516*		*0.810*
**Pulmonary hypertension**						
No	48	265.56 [68.27-903.1]	26	118.3 [21.3-815.3]	22	438.8 [232.7-990.9]
Yes	16	75.4 [5.7-312.1]	8	12.3 [4.2-66.4]	8	154.2 [75.4-576.4]
P value		***0.016***		***0.026***		*0.071*

LVEF: left ventricular ejection fraction. Median values given in pg KIM-1/mg total protein.

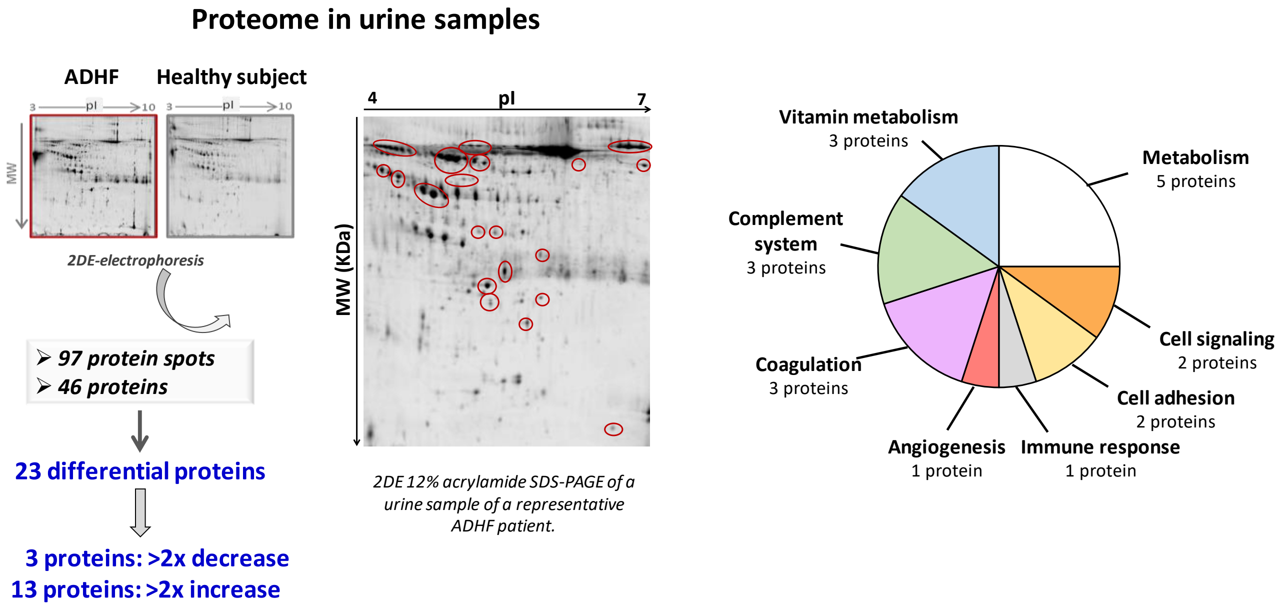

Supplementary Figure I: Distribution of differential proteins detected in 2-dimension electrophoresis gels (left), and on the right are the differential proteins distributed according to their different funcions.

A

Vitamin D binding-protein – Peptide mass fingerprint

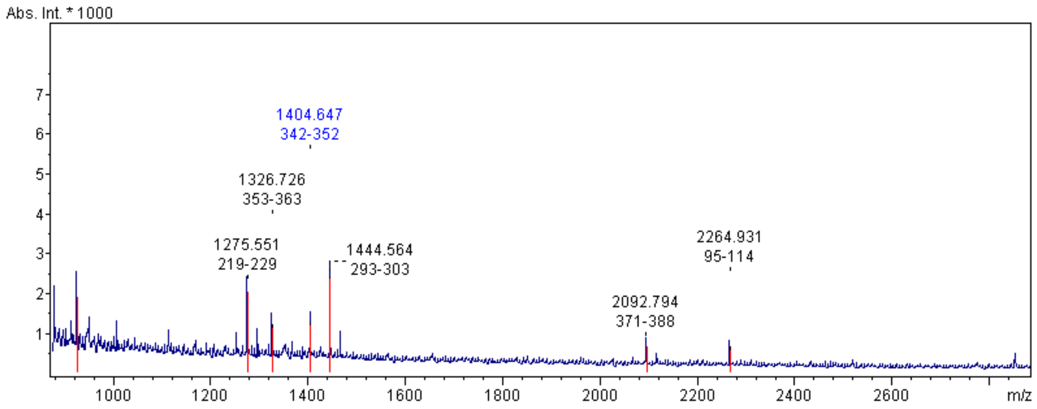

D:\Data\2016-016\H18_OrinaP_047\0_H18\1\1SRef\pdata\1\1r
Unmatched

Peaks	Meas. M/z	Calc. MH+	Int.	Dev.(Da)	Dev.(ppm)	Range	P	Sequence
peak 1	926.360	-	1.880.742	-	-		-	

Vitamin D-binding protein OS=Homo sapiens GN=GC PE=1 SV=1 VTDB_HUMAN
Digest Matches (Score: 94.10)
Modifications: Global: Carbamidomethyl (C), Optional: Oxidation (M),
Search Parameter: Charge=1+, MS Tol.: 50.000000 ppm , MSMS Tol.: 0.500000 Da, Trypsin , Mascot 2.3.0 , SwissProt SwissProt_57.15.fasta

Peaks	Meas. M/z	Calc. MH+	Int.	Dev.(Da)	Dev.(ppm)	Range	P	Sequence
peak 2	1.275.551	1.275.567	2.007.073	-0.016	-12.783	219 - 229	0	VCSQYAAYGEK 2: Carbamidomethyl (C)
peak 3	1.326.726	1.326.753	1.101.192	-0.027	-20.443	353 - 363	1	RTHLPEVFLSK
peak 4	1.404.647	1.404.683	1.175.201	-0.036	-25.612	342 - 352	1	VMDKYTFELSR 2: Oxidation (M)
peak 5	1.444.564	1.444.583	2.351.746	-0.019	-13.309	293 - 303	1	NSKFEDCCQEK 7: Carbamidomethyl (C) 8: Carbamidomethyl (C)
peak 6	2.092.794	2.092.826	664.937	-0.031	-14.945	371 - 388	0	SLGECCDVEDSTTCFNAK 5: Carbamidomethyl (C) 6: Carbamidomethyl (C) 14: Carbamidomethyl (C)
peak 7	2.264.931	2.264.937	558.952	-0.006	-2.806	95 - 114	0	SCESNSPFPVHPGTAECCTK 2: Carbamidomethyl (C) 17: Carbamidomethyl (C) 18: Carbamidomethyl (C)

10	20	30	40	50	60	70	80	90	100	110	120
MKRVLVLLLA	VAFGHALERG	RDYEKNKVCK	EFSHLGKEDF	TSLSLVLYSR	KFPSGTFEQV	SQLVKEVVSL	TEACCAEGAD	PDCYDTRTSA	LSAKSCESNS	PFPVHPGTAE	CCTKEGLERK
130	140	150	160	170	180	190	200	210	220	230	240
LCMAALKHQP	QEFPTYVEPT	NDEICEAFRK	DPKEYANQFM	WEYSTNYGQA	PLSLLVSYTK	SYLSMVGSCC	TSASPTVCFL	KERLQLKHLS	LLTTLSNRVC	SQYAAYGEKK	SRLSNLIKLA
250	260	270	280	290	300	310	320	330	340	350	360
QKVPTADLED	VLPLAEDITN	ILSKCCESAS	EDCMAKELPE	HTVKLCDNLS	TKNSKFEDCC	QEKTAMDVFV	CTYFMPAAQL	PELPDVELPT	NKDVCDPGNT	KVMDKYTFEL	SRRTHLPEVF
370	380	390	400	410	420	430	440	450	460	470	480
LSKVLEPTLK	SLGECCDVED	STTCFNAKGP	LLKKELSSFI	DKGQELCADY	SENTFTEYKK	KLAERLKAKL	PDATPKELAK	LVNKRSDFAS	NCCSINSPPL	YCDSEIDAEL	KNIL

B

Vitamin D binding-protein – MS/MS peak 1444.5

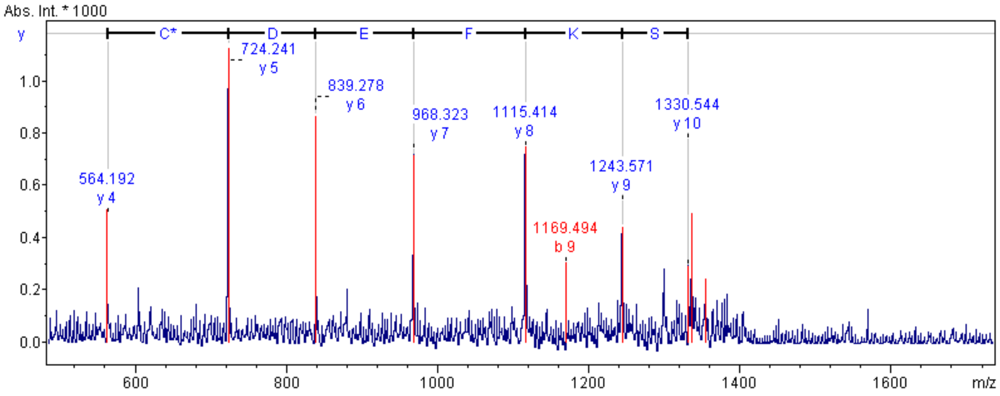

D:\Data\2016-016\H18_OrinaP_047 LIFT1444\0_H18\1\1444.5600.LIFT\1SRef\pdata\1\1r
Vitamin D-binding protein OS=Homo sapiens GN=GC PE=1 SV=1 VTDB_HUMAN
Digest Matches (Score: 61.53)
Modifications: Global: Carbamidomethyl (C), Optional: Oxidation (M),
Search Parameter: Charge=1+, MS Tol.: 50.000000ppm, MSMS Tol.: 0.700000Da, Lys-C ,Mascot 2.3.0, SwissProt SwissProt_57.15.fasta

	Calc. MH+	Meas. Mr	Calc. Mr	Dev.(Da)	Score	MascotScore	Sequence
MSMS 1	1.444.583	1.443.553	1.443.576	-0.023	117	61	NSKFEDCCQEK 7: Carbamidomethyl (C) 8: Carbamidomethyl (C)

Supplementary Figure II: A) Peptide mass fingerprint of a representative VDBPspot. **B)** MS/MS analysis of peak 1444.5 of a representative VDBP spot.

C

Vitamin D binding-protein – MS/MS peak 1275.5

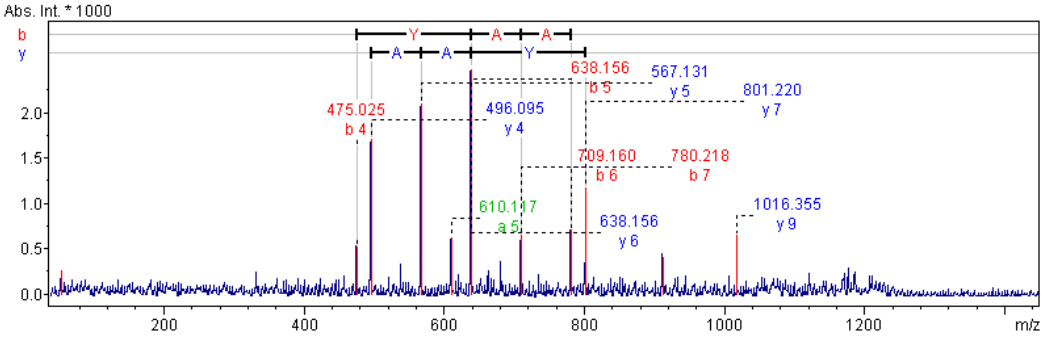

D:\Data\2016-016\H18_OrinaP_047 LIFT1275\0_H18\1\1275.5500.LIFT\1SRef\pdata\1\1r
Vitamin D-binding protein OS=Homo sapiens OX=9606 GN=GC PE=1 SV=2 VTDB_HUMAN
Digest Matches (Score: 41.86)
Search Parameter: Charge=1+, MS Tol.:50.000000 ppm, MSMS Tol.:0.500000 Da, Trypsin , Mascot 2.7.0.7, SwissProt SwissProt_2021_02.fasta
Modifications: Global: Carbamidomethyl (C), Optional: Oxidation (M),

	Calc. MH+	Meas. Mr	Calc. Mr	Dev.(Da)	Score	MascotScore	Sequence
MSMS 1	1.275.567	1.274.543	1.274.560	-0.017	24	41	VCSQYAAYGEK 2: Carbamidomethyl (C)

Supplementary Figure II (continued): C) MS/MS analysis of peak 1275.5 of a representative VDBP spot.

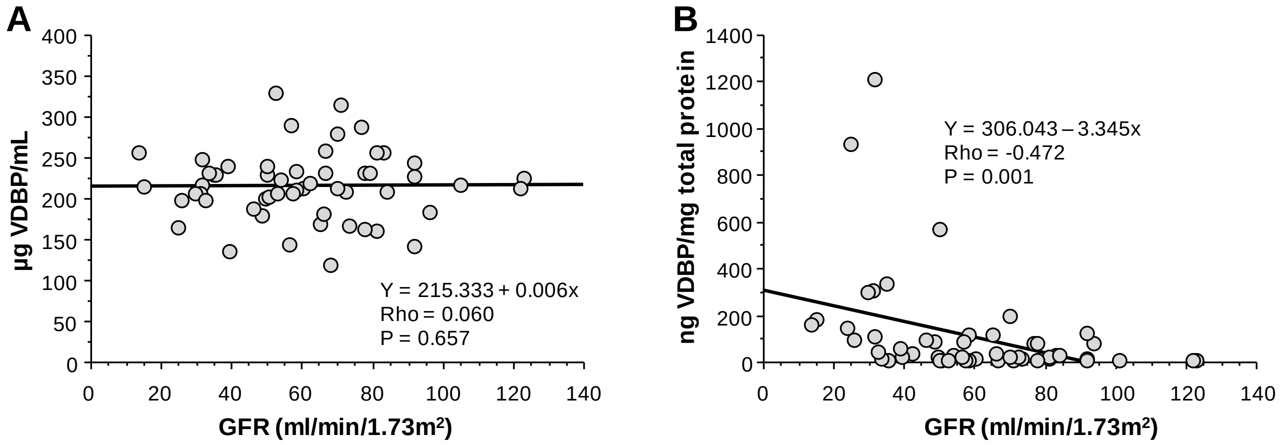

Supplementary Figure III: A) Serum VDBP levels did not correlate with GFR at day 3 of hospitalisation, however, urinary VDBP levels presented a statistically significant correlation with GFR at the same time point.

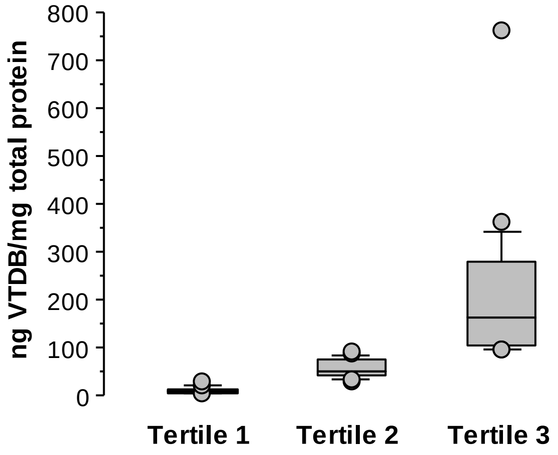

VDBP (ng/mg total protein)
Tertile 1: 8.6 [5.4-13.6]
Tertile 2: 49.2 [41.1-76.0]
Tertile 3: 162.2 [102.2-279.1]

Diabetes patient distribution				
Total ADHF population (N=23)	**7**	**9**	**7**	
NFR (N=9)	3	4	2	**P value** 0.789
RD (N=14)	4	5	5	

Supplementary Figure IV: diabetes distribution according VDBP tertiles in the ADHF population. Values at hospital admission for the total ADHF population and the groups of normal renal function (NRF) and renal dysfunction (RD) patients.

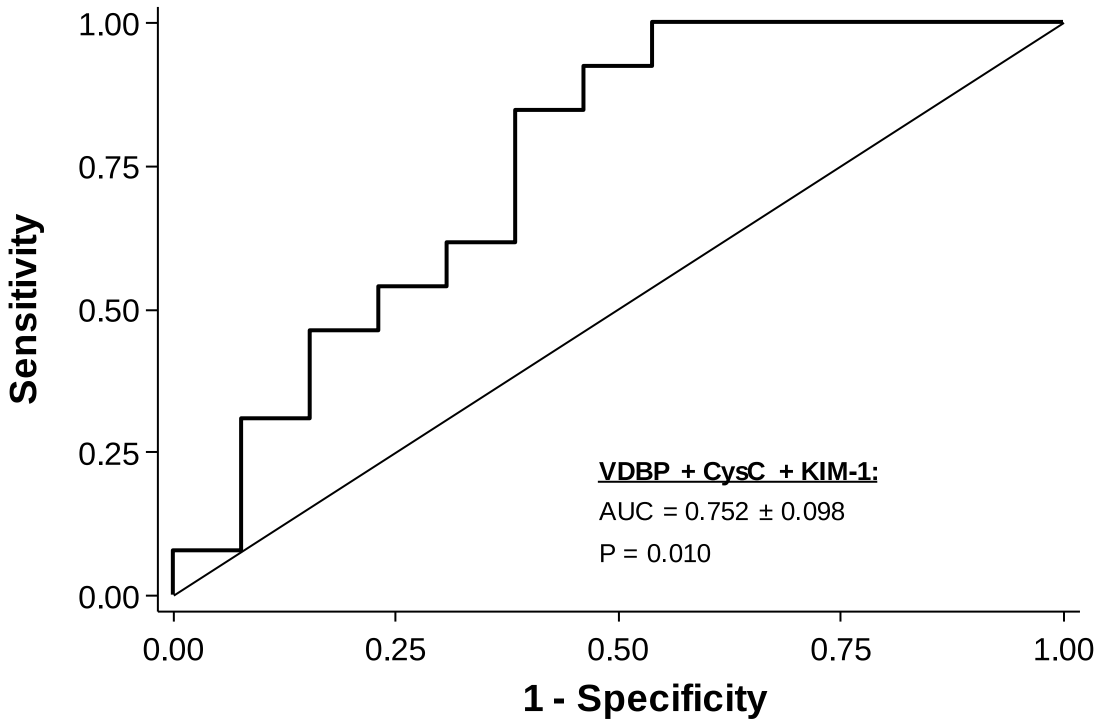

Protein	AUC Area	Error deviation	Lower limit	Upper limit	P-value
VDBP	0.579	0.105	0.374	0.783	*0.446*
CysC	0.476	0.105	0.270	0.682	*0.818*
KIM-1	0.487	0.103	0.284	0.690	*0.901*
CysC + KIM-1 + **VDBP**	0.711	0.089	0.536	0.885	***0.018***
Age	0.618	0.116	0.391	0.846	*0.308*
Age + CysC + KIM-1 + **VDBP**	0.728	0.109	0.515	0.941	***0.036***

VDBP: Vitamin D Binding Protein; CysC: Cystatin C; KIM1: Kidney Injury Molecule 1.

Supplementary Figure V: ROC characteristics of the VDBP, CysC and KIM-1 cluster for early discrimination of patients with renal deterioration compared to age.
